# Assessment of Additive Manufactured IN 625’s Tensile Strength Based on Nonstandard Specimens

**DOI:** 10.3390/ma16144930

**Published:** 2023-07-10

**Authors:** Alexandru Paraschiv, Gheorghe Matache, Mihaela Raluca Condruz, Cristian Dobromirescu

**Affiliations:** 1 Special Components for Gas Turbines Department, Romanian Research and Development Institute for Gas Turbines COMOTI, 220D Iuliu Maniu, 061126 Bucharest, Romania; gheorghe.matache@comoti.ro (G.M.); raluca.condruz@comoti.ro (M.R.C.); cristian.dobromirescu@comoti.ro (C.D.); 2 Section IX-Materials Science and Engineering, Technical Sciences Academy of Romania, 26, Dacia Blvd., 030167 Bucharest, Romania

**Keywords:** additive manufacturing, Inconel 625, sub-sized specimens, tensile properties

## Abstract

The study aimed to evaluate the tensile strength of additively manufactured (AMed) IN 625 using sub-sized test pieces and compare them to standard specimens. Cylindrical round coupons of varying diameters were manufactured along the *Z*-axis using the laser powder bed fusion technique and subjected to heat treatment. The simulation of the alloy solidification predicted the formation of several intermetallics and carbides under equilibrium conditions (slow cooling), apart from the γ phase (FCC). Sub-sized tensile specimens with different gauge diameters were machined from the coupons and tensile tested at ambient temperature. The results showed that sub-sized specimens exhibited lower tensile and yield strengths compared to standard specimens, but still higher than the minimum requirements of the relevant ASTM standard for AMed IN 625. The lower strength was attributed to the “size effect” of the test specimens. Fracture surfaces of the sub-sized test specimens exhibit a mixed character, combining cleavage and microvoid coalescence, with improved ductility compared to standard test pieces. The study highlights the importance of adapting characterization methods to the particularities of manufactured parts, including reduced thicknesses that make sampling standard-size specimens impractical. It concludes that sub-sized specimens are valuable for quality control and verifying compliance with requirements of AMed IN 625 tensile properties.

## 1. Introduction

Since the first process was developed in 1986 [1], additive manufacturing (AM) has gained increasing attention in the manufacturing field, becoming one of the most studied advanced manufacturing technologies. Nowadays, the seven classes of AM processes [2] are used in many fields, such as aerospace [3,4,5,6,7], medicine [8,9,10,11,12], the electronic industry [13,14], and even the food industry [15]. Significant interest was registered for metallic and ceramic complex parts manufactured by AM for high-end industries, such as aerospace. Multiple studies were conducted on metallic materials manufactured by AM, most of them being realized on titanium-based alloys [16,17,18], nickel-based superalloys [19,20,21,22,23,24], cobalt–chromium alloys [25,26,27], and aluminium alloys [28,29,30]. However, current studies validate new materials for AM [31], and the computational methods and programs used for conventionally manufactured alloys are tailored for AM [32,33,34,35,36,37,38,39]. Many standards are applied during the material selection and testing campaigns to produce new parts. Moreover, product acceptance and certification are also conducted by standards. A few years ago, a lack of standards was registered for AM material [40], but recently several standards have been developed that cover the raw material properties, mechanical properties, and characteristics of the finished parts and post-processing methods [41,42,43,44,45,46,47,48].

Despite the advantages of using AM in the industry, AM is still an expensive technology [49]. The metallic and ceramic-based AM processes are the most expensive, starting with the initial machine and materials cost, followed by labour and post-processing costs. Considering the costs implied, minimization of material and time losses are desired in the case of parts manufacturing. Additionally, the implementation of AM for manufacturing of thin-walled and lightweight structures [50], rotating/static components, such as turbine blades [51] or closed rotor blades [52], where critical thickness variations are involved, requires a comprehensive verification of the technology’s capability to produce compliant parts. In this regard, characterization methods must be adapted to the particularities of manufactured parts, which often include reduced thicknesses that make sampling standard-size specimens impractical.

Nowadays, many computational types of research are realized previously in the manufacturing stage to reduce material losses, and a limited number of experimental tests are realized using standard specimens. As many specimens should be manufactured during the testing campaign, a significant quantity of raw material is used, which is expensive and time-consuming. Several studies focused on developing small specimen test techniques (SSTTs) [53,54] that are not limited only to AMed materials to reduce the material and time consumption as much as possible. The SSTs can achieve good consistency with the conventional test and are divided into three categories: similarity (small tensile test—STT and small compression test—SCT), penetration (small punch beam test—SPT and small shear), and semi-penetration (indentation test—IT) [55]. According to Karthik et al. [56], the miniature specimen mechanical testing technology was first used for the material testing of nuclear and non-nuclear industries, the welding industry, and micro- and nanodevices and is defined as a method used to assess the mechanical behaviour of materials using specimens that are considerably smaller than the standard specimen size. This method is also reliable when assessing high-cost materials, including those produced using metal additive manufacturing technology especially as efforts are made to adopt this technology to reduce the cost and time associated with manufacturing aerospace components [57]. Furthermore, the use of miniaturized specimens is relevant for additively manufactured parts with smaller wall thicknesses compared to standard test pieces. Courtright et al. [57] evaluated the viability and effectiveness of several SSTs methods (MSS, SPT, and UTT) for assessing the mechanical properties of Inconel 718 samples produced using Selective Laser Melting technology and found that they can be used to qualify AMed materials and processes.

In the literature, special attention is given to the small tensile specimen test. Dzugan et al. [58] demonstrate the applicability of using miniature Ti6Al4V tensile testing produced using Selective Laser Melting (SLM) and Electron Beam Melting (EBM). Zyl et al. [59] conducted tensile tests on as-built and stress-relieved miniaturised DMLS Ti6Al4V specimens with varying surface qualities, together with full-size specimens, and found that the mini-tensile testing can be used effectively only by applying a correction factor related to the surface roughness. Reddy et al. [60] studied the tensile properties of five alloys (IN 718, CoCrMo, Maraging steel, SS316L, and Ti6Al4V) manufactured by direct metal laser sintering—DMLS based on micro-specimens. It was concluded that tensile properties in an as-built state are close to their counterparts manufactured according to the Standard. The yield strength (YS), ultimate tensile strength (UTS), and elongation of CoCrMo, Maraging steel, and SS316L were reduced by 5%, while the 10% IN 718 and Ti6Al4V were reduced by IN 718 and Ti6Al4V. SSTT viability was also demonstrated by Kumar et al. [61] for three types of conventionally manufactured steels (20MnNiMo55, CrMoV, SS304LN) and three types of specimens by Dongare [62] in the case of wrought and AMed Ti6Al4V, and by Robbins [63] in case of AMed IN 625 and AlSi10Mg. Overall, the results established that SSTT is a viable AM metal quality control technique, but there is a significant requirement for more AM-specific standards. In response to this need, ASTM International is currently developing a standard for miniature tension testing of metallic materials (ASTM WK75901 [64]).

The main objective of this study is to evaluate the tensile strength of the laser-based powder bed fusion (L-PBF) melted IN 625 nickel-based superalloy using sub-sized specimens and the test specimen size impact on the material properties. The specific focus of the study is to investigate the influence of test specimen size on material properties. Other characteristics of the AM IN 625, such as the microstructure features, grain size and the role of grain boundaries, and their influence on tensile test results obtained on sub-sized test specimens, were discussed.

## 2. Materials and Methods

For the current study, a Lasertec 30 SLM machine, which has a total building volume of 300 × 300 × 300 mm (L × l × H), was utilized along with IN 625 metallic powder manufactured by gas atomization and distributed by LPW Technology Ltd. (Runcorn, UK). The raw material’s particle size distribution (PSD) was experimentally determined before the manufacturing campaign started. For the PSD analysis, a Retsch AS 200 Control analytical stainless steel sieve shaker with a volume of 100 × 40 mm (D × H), wire mesh sieves (20–50 μm) and a collector were utilized. Four 50 g powder samples were analysed, extracted from random areas on the building plate using a spatula after completing a job, and were subsequently analysed. The sieving parameters used included a sieving duration of 5 min, an amplitude of 2 mm, and a 60-s interval time. The powder particles’ morphology and fracture surfaces of tensile specimens were investigated by scanning electron microscopy (SEM) using the FEI F50 Inspect SEM (FEI Company, Brno, Czech Republic).

To assess the tensile properties of IN 625 using sub-sized test specimens and the influence of specimen thickness on the mechanical properties, cylindrical round coupons with various diameters were manufactured in a vertical orientation (along *Z*-axis) using the same process parameters. The vertical orientation was selected based on findings from different publications, which indicate that it typically exhibits the lowest tensile strength values for various AMed materials [65,66,67]. The coupons were manufactured using the following process parameters: laser power of 250 W, scanning speed of 750 mm/s laser speed, hatch angles with rotation of 90° between successive layers, a layer thickness of 40 μm, a hatch distance of 0.11 mm, and a building plate maintained a temperature of 80 °C during all manufacturing processes.

Three different types of coupons, each measuring 45 mm in length, were built in the same job along *Z*-axis:

- Cylindrical 10 mm in diameter, the same as the coupons used to manufacture standard specimens;
- Cylindrical 6.5 mm in diameter (to allow machining of M6 thread);
- Profiled coupons with 3.5 mm in diameter to machine the gauge length and 6.5 mm in diameter (at the ends) to machine the M6 thread.

**Figure 1:**
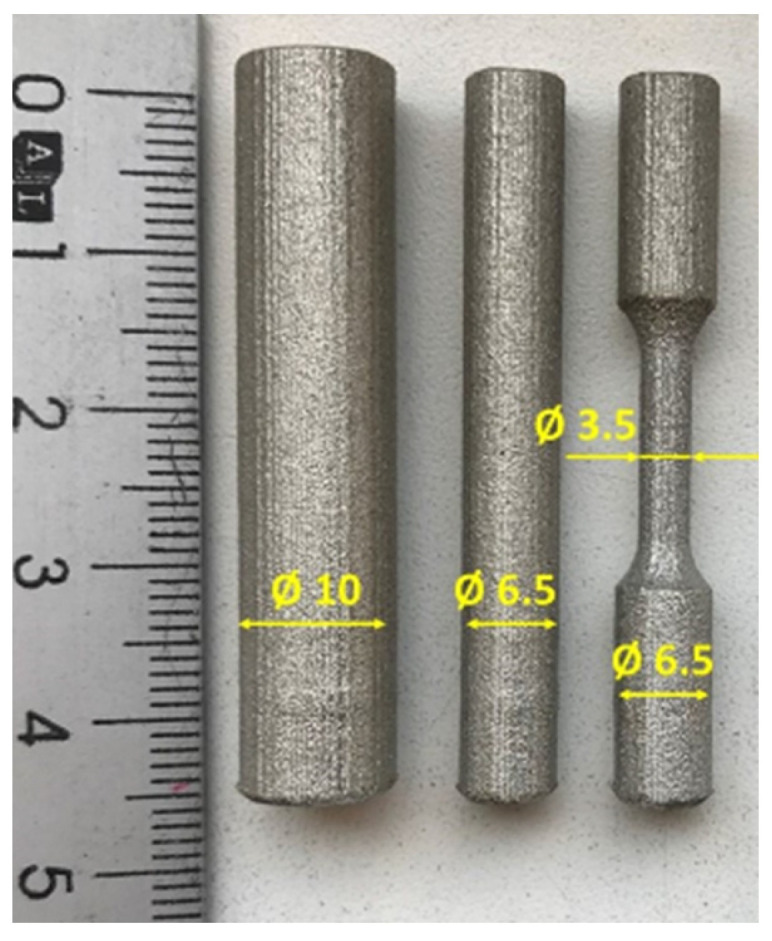
Coupons used for sub-size test specimens’ manufacturing.

The IN625 coupons with 3.5 mm, 6.5 mm and 10 mm in diameter used for sub-sized test specimen manufacturing are presented in Figure 1.

**Figure 2:**
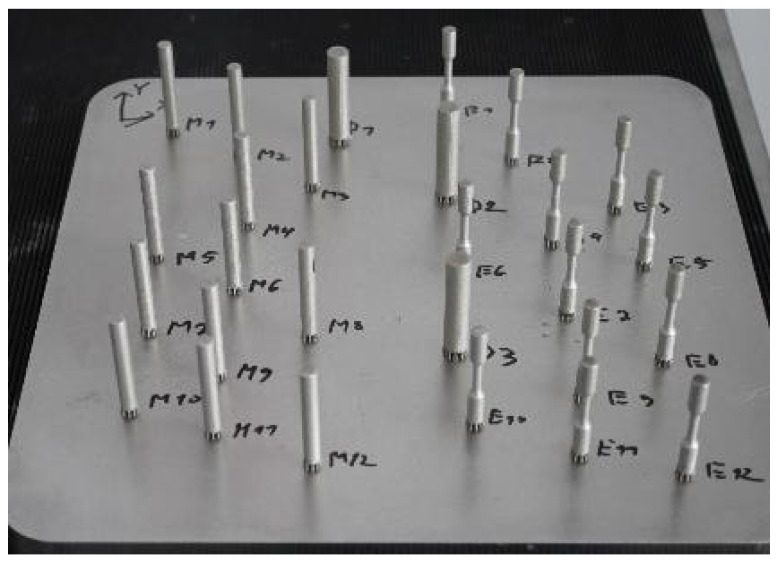
Coupons of different diameters built into one job.

A total of 12 coupons with a diameter of 6.5 mm and 12 profiled coupons with a gauge length area of 3.5 mm were built together in two different jobs. Additionally, a limited number of 3 coupons of 10 mm in diameter were built in one of the jobs. The purpose of manufacturing the 10 mm diameter coupons was only to assess any differences compared to the cylindrical 6.5 mm diameter coupons with 3 mm machined gauge diameter. Figure 2 presents all coupons manufactured in one job.

Before machining, all coupons were heat treated together in the same conditions. The heat treatment conditions of L-BPF IN 625 were chosen by the authors based on previous works [52,68,69]: a stress relieving treatment was applied involving heating from room temperature to 870 °C and holding the temperature for 1 h. Subsequently, an annealing heat treatment was performed by heating the coupons from room temperature to 1000 °C, holding the temperature for 1 h, and then rapidly cooling them in oil (oil quenching).

The material’s microstructural features were studied in an as-built state and after heat treatment. From each set of 12 coupons with a diameter of 6.5 mm and 12 profiled coupons, machined sub-sized test specimens were obtained as described below:

- From the cylindrical 6.5 mm diameter coupons: 7 test specimens with a gauge diameter of 3 mm (referred to as D3/D6.5) and 5 test specimens with 2.5 mm gauge diameter (referred to as D2.5/D6.5);
- From the profiled coupons with a 3.5 mm diameter in the gauge area: 7 test specimens with a gauge diameter of 3 mm (referred to as D3/D3.5) and 5 test specimens with a 2.5 mm gauge diameter (referred to as D2.5/D3.5).

**Figure 3:**
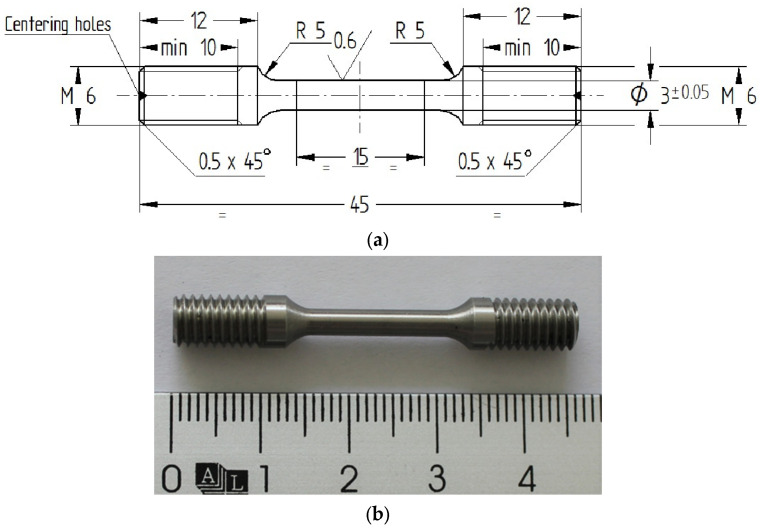
Sub-sized test specimen’s dimensions (3 mm gauge diameter). (**a**) Specimen dimensions; (**b**) Specimen machined.

A round sub-sized test specimen with a machined 3 mm gauge diameter is shown in Figure 3.

The surface roughness of the tensile specimens after machining was determined using the MarSurf PS 10 mobile roughness measuring instrument (Mahr Inc., Providence, RI, USA).

The results of the tensile testing using sub-sized tensile specimens were compared with the average result obtained using standard specimens with a 5 mm gauge diameter manufactured on the *Z*-axis (UTS of 834 ± 6.4 MPa, YS of 542 ± 5.4 MPa, reduction in the area—RA—54 ± 1.6%, elongation 49.1%), previously tested by the authors [68]. To ensure that the testing force of the sub-sized test specimens was not less than 10% of the load cell (i.e., >5 kN) and considering the tensile strength of the material in the Z-direction, the gauge length diameter of the test specimens was restricted to 3 mm.

Test pieces with a gauge length of 15 mm and 3 mm in diameter fulfil the *Lo* requirements of both ASTM E8/E8M-22 [70] and EN ISO 6892-1:2009 [71] and the proportionality to standard test specimens. Moreover, threaded ends were utilized to secure the test pieces into the testing device, ensuring that the specimens would not slip out of the machine grips. Additionally, even if the estimated maximum testing force falls below 5 kN, sub-sized test specimens with a reduced gauge diameter of 2.5 mm were also manufactured and tested.

Tensile testing at room temperature of the sub-size test specimens was performed according to EN ISO 68921-1:2009 [71] using the electromechanical universal testing machine, Instron 3369 Dual Column Testing System (Instron, Norwood, MA, USA), with a load cell of 50 kN. An Instron 10 mm gauge length Dynamic Extensometer, Class 0.5%, was used. All test specimens were tested using two strain rates over the parallel length. Initially, a strain rate of eLc = 0.00025 s^−1^ was used while the extensometer was attached to the specimen. Once the yield strength (YS) was recorded, the extensometer was removed, and a strain rate of eLc = 0.0067 s^−1^ was used until the end of the test.

**Figure 4:**
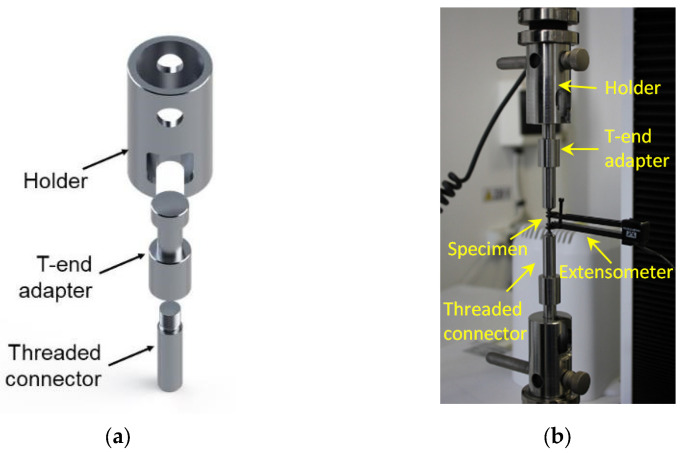
Tensile testing of sub-sized specimens: (**a**) Fastening device and (**b**) Machine set-up for tensile testing.

For the tensile test, a particular device was designed and manufactured to fix the test specimens and to allow the extensometer to be fitted between machine grips. Figure 4 illustrates the typical set-up of the machine and the fixture device used for tensile testing of the sub-sized test specimen.

The fractographic analysis of the specimens subjected to tensile testing was performed by SEM using the FEI F50 Inspect microscope (FEI Company, Brno, Czech Republic), equipped with an energy dispersive X-ray spectrometer (EDS) EDAX APEX 2i with SDD Apollo X detector (EDAX Inc., Ametek MAD Mahwah, NJ, USA). Fixing heads of the tensile specimens were cut after the tensile test was performed, and they were metallographically prepared by grinding, polishing, and etching with Aqua Regia reagent.

Grain size measurements were realized using the intercept method on 100× magnification optical micrographs captured using the optical microscope with camera Axio Vert.A1 MAT (Carl Zeiss Microscopy GmbH, Jena, Germany). The optical images were processed to emphasize the microstructural features using the Scandium software (version 5.2, Olympus Soft Imaging Solutions GmbH, Münster, Germany), and the average grain size was determined based on measurements made on five light optical microscopy images. The fine carbides precipitated at the grain boundaries were evaluated qualitatively using SEM analysis and the element distribution maps (EDS mapping). Phase predictions were realized using Pandat™ software (2021, CompuTherm LLC, Middleton, WI, USA), PanSolidification Module with the PanNi_2021 database. By entering the chemical composition of IN 625 in this module, the software automatically calculates phase fractions as a function of temperature based on its database.

## 3. Results

### 3.1. Powder Characteristics

**Figure 5:**
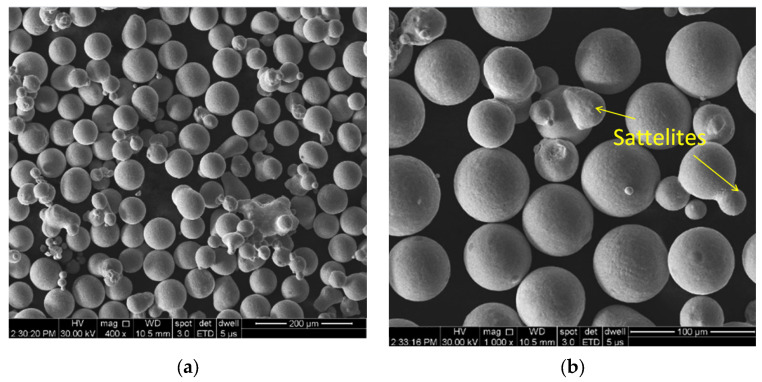
SEM images of IN 625 powder with spherical particles and particles with satellites. (**a**) image at 400×; (**b**) image at 1000×.

The experimental determined PSD of the raw material was D10 = 20 μm, D50 = 30 μm, and D90 = 39 μm. The powder used is characterized mainly by smooth spherical particles, but particles with satellites can also be observed as is shown in Figure 5a,b.

### 3.2. Tensile Test Results

#### 3.2.1. Sub-Sized Test Specimens with 3 mm Gauge Diameter

The results of tensile tests at room temperature of all specimens with 3 mm gauge diameter, machined from the two types of coupons, and manufacturing jobs are presented in Table 1, Table 2, Table 3 and Table 4. Tensile and yield strength, reduction in area and elongation after fracture were measured according to ISO 6892-1:2009 [71].

#### 3.2.2. Sub-Sized Test Specimens with 2.5 mm Gauge Diameter

The results of tensile tests of all test specimens with 2.5 mm gauge diameter machined from the two types of coupons and manufacturing jobs are presented in Table 5, Table 6, Table 7 and Table 8.

The analysis of the results presented in Table 1, Table 2, Table 3, Table 4, Table 5, Table 6, Table 7 and Table 8 indicates differences in the tensile properties of sub-sized specimens compared to standard specimens. In general, small specimens can retain the same mechanical characteristics as conventional specimens if there are similarities in the geometry and dimensions of structural elements (grains, grain boundaries, second phase, inclusions), deformation mode and stress state during loading, specimen geometry, test fixtures, and clamps [55].

Therefore, this study aims to further investigate additional characteristics of the AMed IN 625. These characteristics include the fractographic features of the specimens, surface roughness, developed microstructure, grain size, grain boundaries, and their influence on tensile behaviour.

### 3.3. Fractographic Analysis

**Figure 6:**
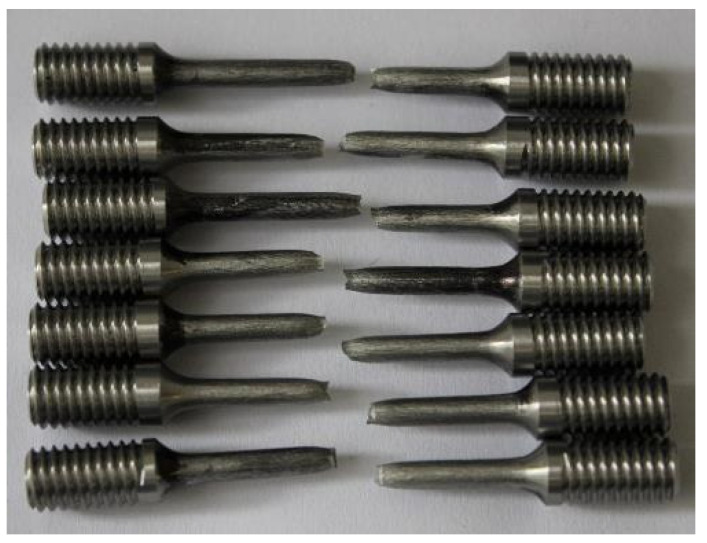
The typical cup-con shape of the necked region of the 3 mm gauge diameter test specimens (top—M1 test specimen; down—M7 test specimen).

The fracture surface of the test specimens after the tensile test shows a cup-cone shape of the necked region. Figure 6 presents the 3 mm gauge diameter sub-sized test specimens after fracture (manufactured during the second job).

**Figure 7:**
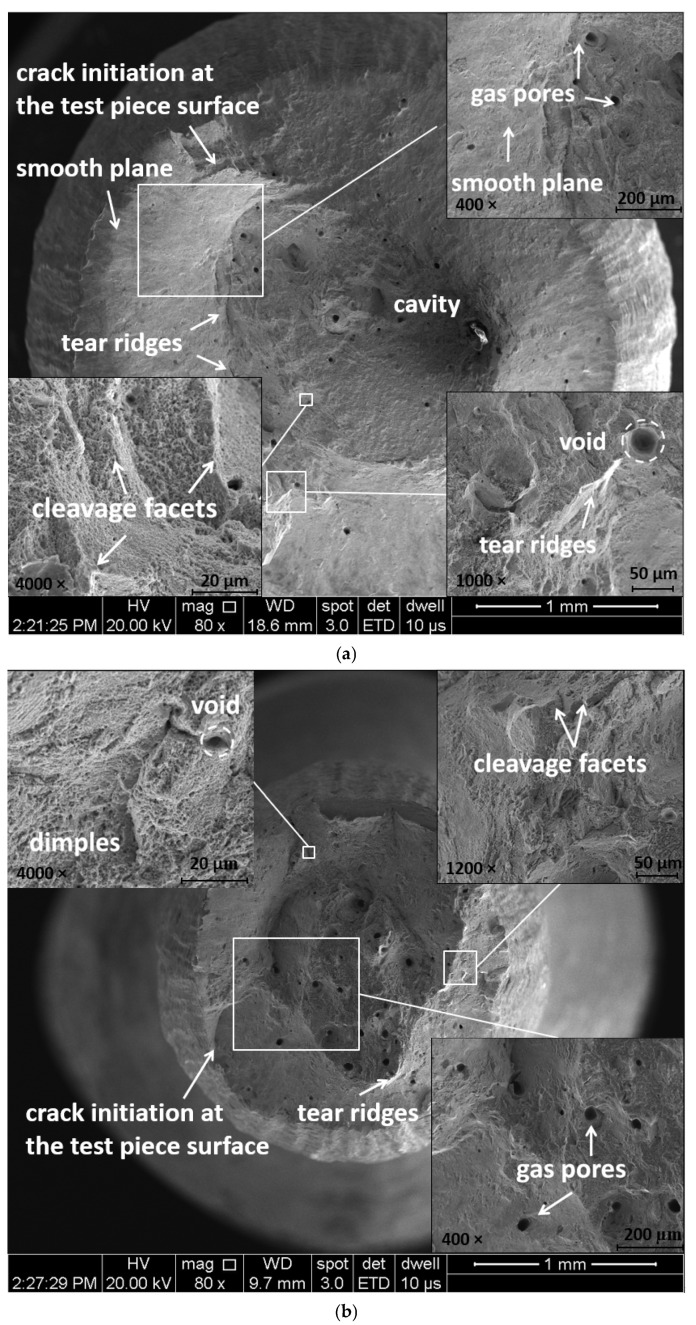

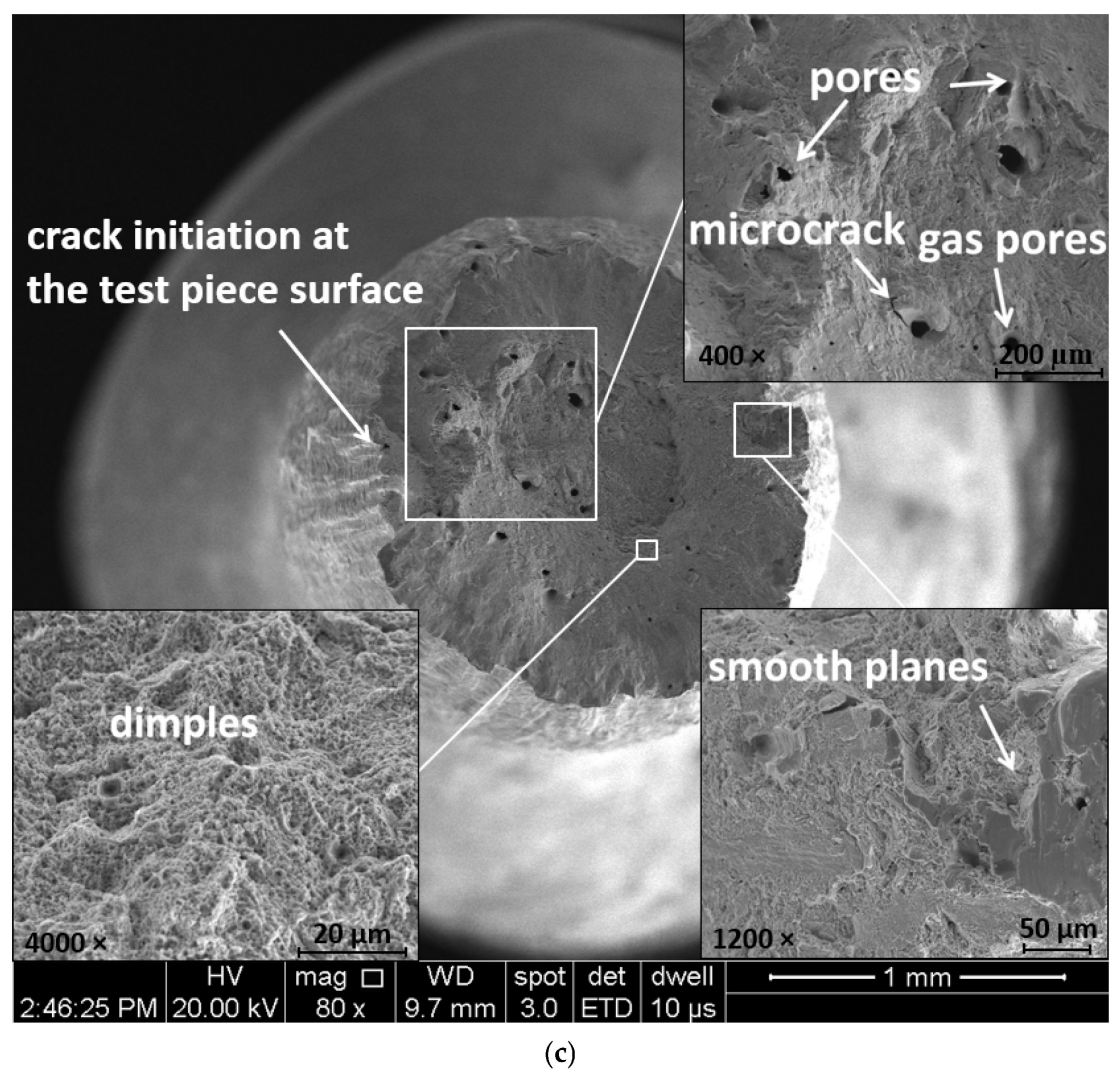
SEM fracture surfaces with details at higher magnification showing the mixed fracture mode, finer microvoids, and porosities of the test pieces: (**a**) 5 mm gauge diameter, (**b**) 3 mm gauge diameter, (**c**) and 2.5 mm gauge diameter.

SEM investigation of the fracture surfaces shows a cup-and-cone shape of the necking region and a dimple rupture. The fracture mode is mixed (ductile and brittle fracture), showing cleavage facets and microvoid coalescence, resulting in a dimpled appearance, cleavage facets and opened-up pores. Standard specimens manufactured using the same IN 625 powder and previously tested by the authors in another work [68] showed similarities in terms of fracture surfaces and material defects. Pore formation or growth during heat treatment, known as thermally induced porosity, is attributed to the local plastic deformation caused by the entrapped gas expansion during heating [72]. These pores can act as stress concentrators, leading to the initiation and ultimately the propagation of fracture cracks under tensile loading. Figure 7a–c presents the fracture surfaces of a standard specimen and sub-sized test specimens, revealing distinctive features of the fracture mode. While all specimens exhibit fracture surfaces with a mixed character resulting from a combination of cleavage and microvoid coalescence, the sub-sized test specimens exhibited higher ductility compared to the standard specimens, as shown by the elongation values in Table 1, Table 2, Table 3, Table 4, Table 5, Table 6, Table 7 and Table 8. This behaviour can be attributed to grain boundary strengthening and may explain the higher elongation after fracture observed in the sub-sized test specimens.

### 3.4. Sub-Sized the Specimens’ Roughness

Some authors reported that with the miniaturization of specimens, surface roughness plays an important role in determining the effective cross-section of the specimen. Kashaev et al. [73] proposed a method for correcting the cross-section of sub-sized specimens when analysing the strength of conventional manufactured IN 625, IN 718, and Ti-6Al-4V in comparison with standard specimens, while Bradley et al. [74] investigated the tensile performances of additively manufactured stainless steel on miniature tensile bars. Both papers considered the correction of the effective cross-section by taking into account the surface roughness. This correction involved reducing the measured cross-section area of the specimens based on the surface roughness measurements of the test specimens. While this method is effective for rough surfaces, such as the as-built sub-sized specimen’s surfaces, for machined specimens’ simple correction due to roughness is ineffective.

Tensile test specimens used in this study were prepared by turning and finished grinding, resulting in a measured surface roughness of Ra = 0.4 μm and Rz = 4.1 μm. To account for the roughness of the measured surface, a correction factor was introduced to calculate the effective test specimen gauge diameter (Equation (1)). Subsequently, the tensile strength of the specimens was recalculated based on this correction.

*d_eff_* = *d*_0_ − 2·*Rz*, (1)

where *deff* is the effective diameter of the specimen, *d*_0_ is the nominal diameter of the specimen and *Rz* is the average value of the heights of the five highest-profile peaks and the depths of the five deepest valleys on the surface.

The results of tensile tests measured by machine for *d*_0_ and recalculated for *d_eff_* are summarized in Table 9 and Table 10 for specimens with 2.5 mm and 3 mm gauge diameter, machined from 6.5 mm in diameter coupons. Minor differences, less than 1%, were recorded between the test pieces’ measured and recalculated tensile strengths. The tensile strength increase in the 2.5 mm gauge diameter test pieces is only 0.65%. Increasing the test specimens’ gauge diameter from 2.5 mm to 3 mm, the influence of the correction is even smaller (average 0.55%).

The test pieces machined from the profiled coupons show the same insignificant increase for both gauge diameters.

### 3.5. Microstructural Characteristics of the Test Specimens

**Figure 8:**
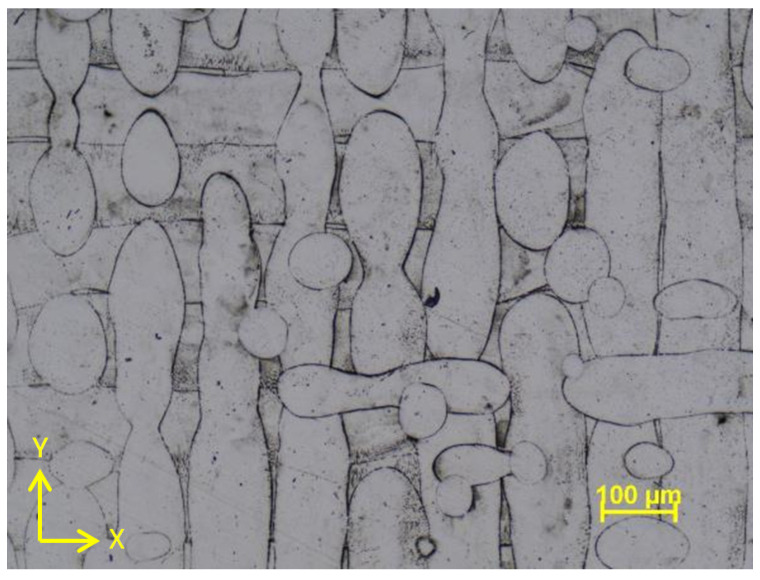
Light optical micrograph of the AMed IN 625 in cross-section (parallel to X-Y plane) showing the melt tracks (100×).

In as-built conditions, the material exhibits anisotropic microstructural features. Therefore, heat treatment is an important step after building a part to eliminate as much as possible anisotropy and generates a more isotropic, equiaxed structure by recrystallization. Figure 8 presents a light optical micrograph of the as-built IN 625 in a section parallel to the building X-Y plane, taken in a cross-section of a 6.5 mm coupon built-in vertical position (along the machine Z axis). The sections are perpendicular to the tensile test-loading direction.

**Figure 9:**
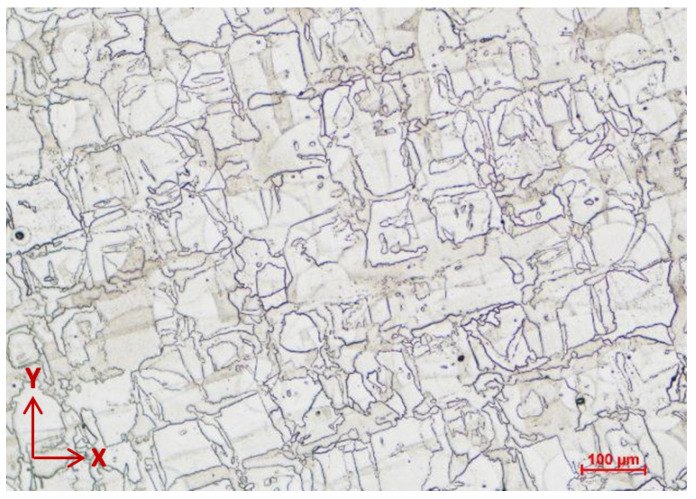
Light optical micrograph of the AM IN 625 in cross-section (parallel to X-Y plane), recrystallized microstructure after annealing (100×).

In the as-built state, the microstructure of AMed IN 625 predominantly comprises columnar primary dendrites. This dendritic structure is maintained when the alloy is annealed below 1000 °C [75]. After the standard annealing heat treatment at 1000 °C, the investigated material shows a recrystallized microstructure. However, above this temperature, recrystallization occurs, and it is completed when annealing is performed at 1200 °C [75]. Figure 9 shows this microstructure for a 6.5 mm diameter specimen in the same sectioning direction as that of the as-built state.

**Figure 10:**
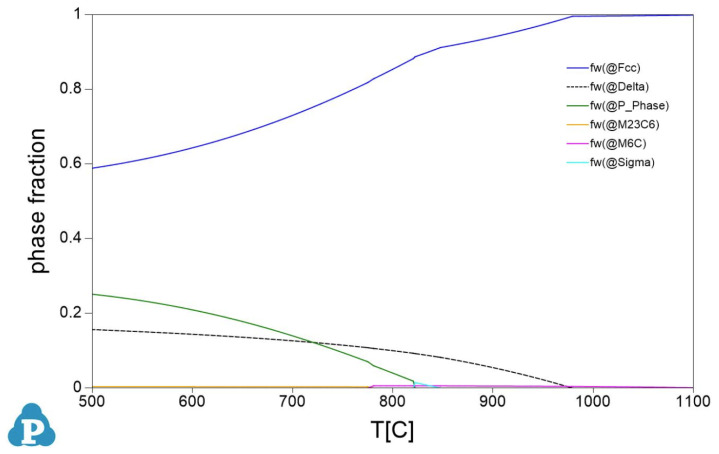
Prediction of phase fractions during the solidification and cooling of IN 625 alloy under equilibrium conditions.

Due to IN 625 chemistry (no content of γ’ forming elements, i.e., Al, Ti), the material is not a precipitation-hardening alloy. The role of the annealing heat treatment is only to generate an equiaxed, recrystallized microstructure. The simulation of the alloy solidification using Pandat™ software in multiphase, multicomponent systems predicts under equilibrium conditions (slow cooling) the formation of several intermetallics and carbides apart of γ phase (FCC)—Figure 10.

However, this is not the case with AM solidification, which involves high cooling rates (10^5^–10^6^ K/s) and suppresses the formation of phases by transforming the solid state at low temperatures. The only high-temperature minor phases in the alloy are carbides that meet thermodynamic conditions to form by liquid state reaction. These primary carbides (M6C) transform further during cooling in the solid state into M23C6-type carbides. This finding is in agreement with other data determined by thermodynamic modelling and STEM-EDS obtained by Maciol et al. [76] who investigated the precipitates in L-PBF Inconel 625 subjected to high-temperature annealing.

**Figure 11:**
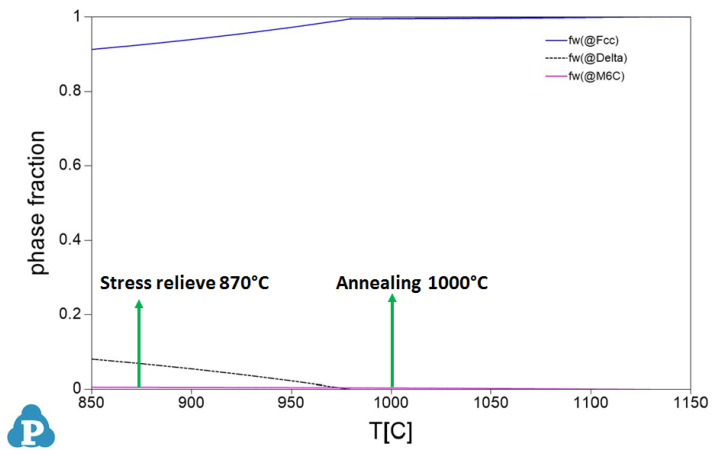
Prediction of phase fractions as a function of temperature in AMed IN 625.

Figure 11 presents the phase fractions that form in AM IN 625 alloy. The stress-relieve and annealing heat treatment temperatures used in this study are overprinted.

**Figure 12:**
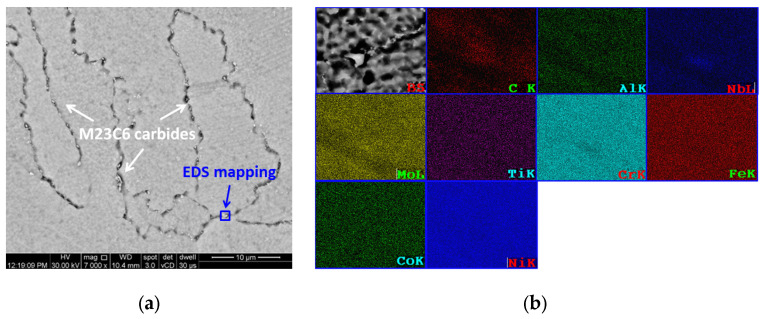
SEM analysis of the AM IN 625 in cross-section (parallel to X-Y plane) after annealing: (**a**) backscattered electron image showing a network of fine carbides precipitated at the grain boundaries and (**b**) EDS mapping of microarea with carbides.

The Delta phase, as shown by a dotted line in Figure 11, does not form directly during the solidification process of AMed IN 625. Instead, this phase, which has the potential to detrimentally affect mechanical properties, can only develop after extended periods of exposure at temperatures below 975 °C. The annealing temperature exceeding 975 °C results in the solutionizing of the Delta phase. In the case of AMed IN 625, an annealing temperature over 1000 °C is utilized, accompanied by fast cooling to room temperature preventing the formation of the Delta phase, but the carbides are still present. After annealing, the structure of the alloy is expected to primarily consist of the γ phase, with only a small fraction (estimated to be less than 1%) of carbides precipitated at the grain boundaries. In the heat-treated state, the investigated AM IN 625 alloy exhibits recrystallized grains of γ phase and a fine network of precipitated carbides that strengthen the grain boundaries. Figure 12a presents the alloy’s SEM backscattered electron image showing a fine carbides network precipitated at the grain boundaries. Additionally, Figure 12b shows the EDS mapping of the microarea with carbides. No Delta phase was observed in the investigated section. The image was captured in a cross-section of a 6.5 mm diameter coupon built in the Z-direction of the machine.

### 3.6. Grain Size and Grain Boundaries

Determining the impact of the microstructure on the mechanical performance is crucial for comprehending the material behaviour in tension, particularly when sub-sized test specimens are used. According to Lorenzo et al. [77], the miniaturization of specimens induces a “scaling effect” altering material behaviour at the microscale in comparison to the macroscale. This effect can involve factors such as grain size and their quantity in the cross-section, anisotropy, micro-structural and chemical inhomogeneity and residual stress. In addition, other characteristics of the AM alloy microstructure need to be taken into account, including grain size, the role of grain boundaries, and their influence on small-size test pieces’ mechanical properties.

As mentioned previously, the IN 625 is not a precipitation-hardening alloy. Therefore, the annealing heat treatment generates recrystallized grains and precipitated carbides that ensure grain boundaries stiffness and play an important role in the deformation mechanisms of IN 625 alloy. From this perspective, the results of the tensile test on sub-sized test specimens were analysed in connection with the grain size and grain boundary characteristics of the AMed IN 625 alloy. Measurements of grain size using the optical microscope on 6.5 mm diameter and 3.5 mm profiled specimens revealed slightly different grain size values. The average grain size of 55 μm was obtained for the 6.5 mm specimens, while the average value of 63 μm was obtained for the 3.5 mm specimens. The difference is explained by the different thicknesses of the specimens that have been treated together for the same duration. However, when analysing the results obtained on specimens with different diameters (2.5 mm and 3 mm) from the same type of coupon (6.5 mm or 3.5 mm) having the same grain size, it is obvious that the lower the gauge diameter, the lower the recorded strength.

From this perspective, when comparing specimens with the same grain size, a smaller diameter of the test specimen means a smaller number of grains in the cross-section. As a result, the grain boundary density is lower, which limits the resistance to dislocation movement and, consequently, deformation under stress. This behaviour helps explain why sub-sized specimens exhibit slightly higher elongation compared to the standard specimen, which has a larger number of grains in its cross-section.

To assess the relationship between grain boundaries and the strength of the sub-sized pieces, a dimensionless parameter was used. This parameter establishes a connection between the total length of the grain boundaries in a section and the cross-section of the test piece, as depicted in Equations (2)–(5).

*Dr* = *lgr*/√*So* (2)

*lgr* = 2·(*n* + 1)·*Ssl* (3)

*Ssl* = *r*√*π* (4)

*n* = *Ssl*/*Gs* (5)

where *Dr* is the dimensionless parameter, *lgr* is the total length of the grain boundaries in the test piece cross-section, *So* is the test piece cross-section area, *Ssl* is the square side length (calculated from the circle’s area), *r* is the radius of the sample, *n* represents the number of grains, and *Gs* is the average grain size (measured from optical images).

The measurements and results obtained on sub-sized test specimens are presented in Table 11, considering round shape grains for simplification.

**Figure 13:**
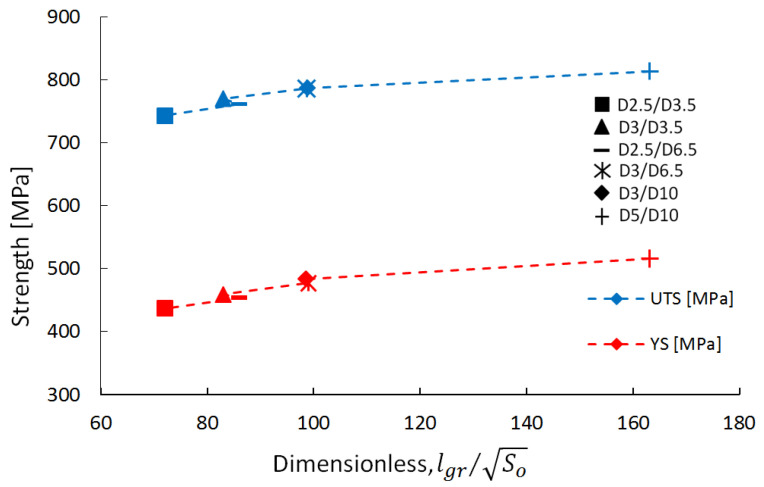
Sub-sized test specimens’ strength as a function of the dimensionless parameter.

This study aimed to establish a correlation between sub-sized test piece strength and the dimensionless microstructural parameter. The results revealed that as the number of grains of the same size in the test specimen cross-section (higher test specimen gauge diameter) increases, the strength also increased. This behaviour follows the trends shown in Figure 13. Due to the incomplete recrystallization of the AMed IN 625 material in comparison to the conventionally manufactured material, accurately measuring the grain size can be challenging. As a result, this approach holds primarily qualitative value rather than precise quantitative.

## 4. Discussion

**Figure 14:**
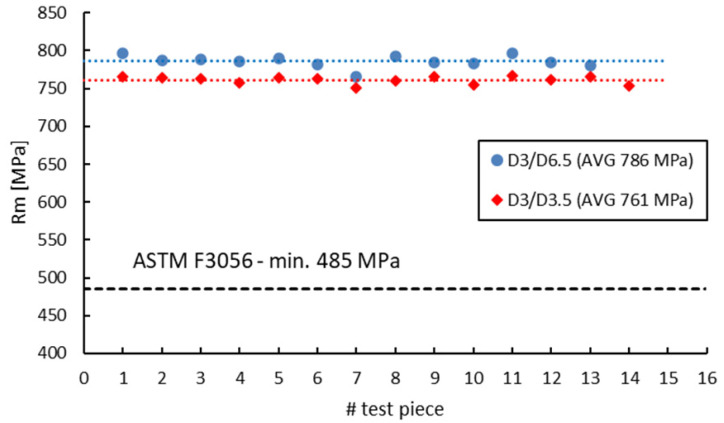
Tensile strength of 3 mm gauge diameter test specimens.

**Figure 15:**
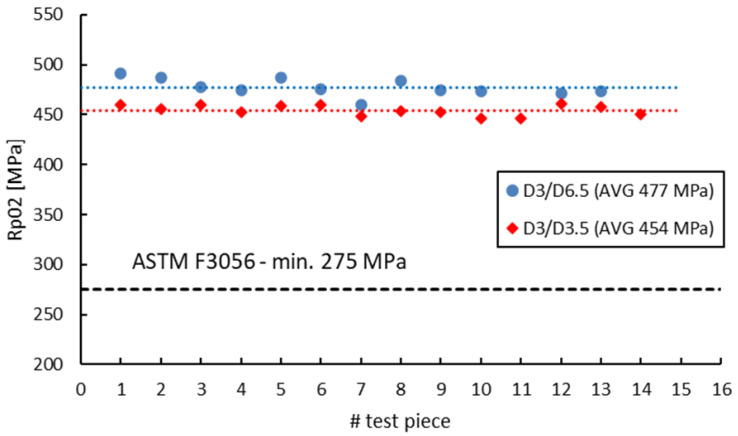
Yield strength of 3 mm gauge diameter test specimens.

**Figure 16:**
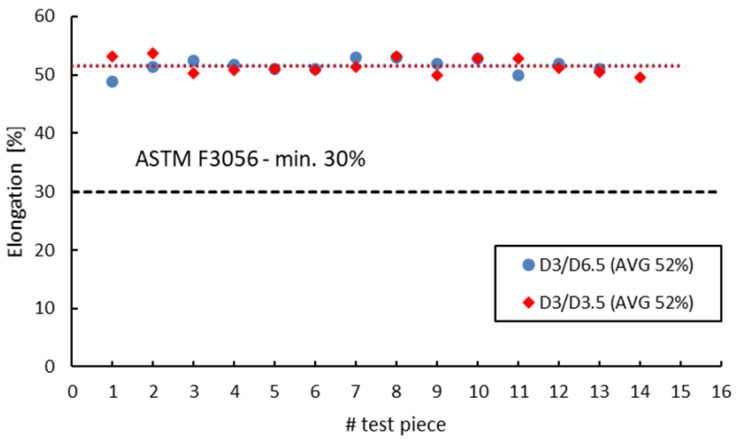
Elongation after fracture of 3 mm gauge diameter test specimens.

**Figure 17:**
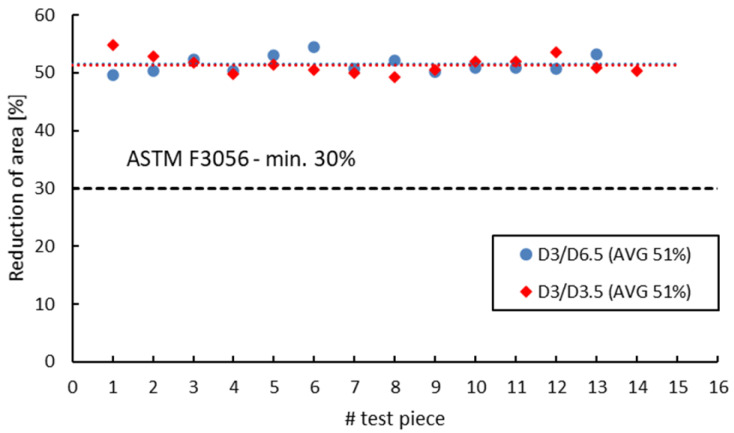
Reduction in area of 3 mm gauge diameter test specimens.

ASTM F 3056 [41] provides reference data for AMed IN 625 mechanical properties checked by tensile testing on standard test pieces. However, when manufacturing parts with smaller wall thicknesses compared to the standard tensile test specimens, a suitable and reliable method for assessing material properties on nonstandard witness test specimens is required. Neither ASTM E8/E8M-09 [70] nor EN ISO 6892-1:2009 [71] do not give limitations to the minimum size of the tensile test pieces. Instead, both standards refer to “small-size” or “sub-sized” flat or round test Specimens Proportional to the Standard. ASTM specifies that alternative specimens can be utilized as long as the ratio between the gauge length and diameter is maintained at 4-to-1 or 5-to-1. On the other hand, EN ISO 6892-1:2009 [71] recommends the use of proportional test pieces, where the gauge length should be *Lo* = 5.65√*S*, where *S* is the cross-sectional area, but not less than 15 mm. However, regardless of the sizes, geometry or standards used, the sub-sized tensile specimens must be validated and qualified against standard specimens. Figure 14, Figure 15, Figure 16 and Figure 17 present a comparison of the tensile test results obtained on 3 mm gauge diameter sub-sized tensile test specimens machined from coupons with different diameters, 6.5 mm and 3.5 mm, respectively. Test specimens no. 1 to 7 of both series were built together in the first job, and test specimens no. 8 to 14 were built together in the second job. The black dotted lines in the figures represent the minimum mechanical property requirements according to ASTM F3056 [41], while the red and blue dotted lines represent the average values of all test pieces.

As seen in Figure 14, Figure 15, Figure 16 and Figure 17, the sub-sized test specimens manufactured from 6.5 mm diameter coupons exhibit higher tensile strength and yield strength than the test specimens manufactured from profiled coupons. Both elongation after fracture and reduction in area present consistent (similar) average values. Nevertheless, when compared to the standard test specimens, all 3 mm diameter sub-sized test specimens exhibit lower tensile strength values, yield strength, and reduced area (refer to Table 12).

**Figure 18:**
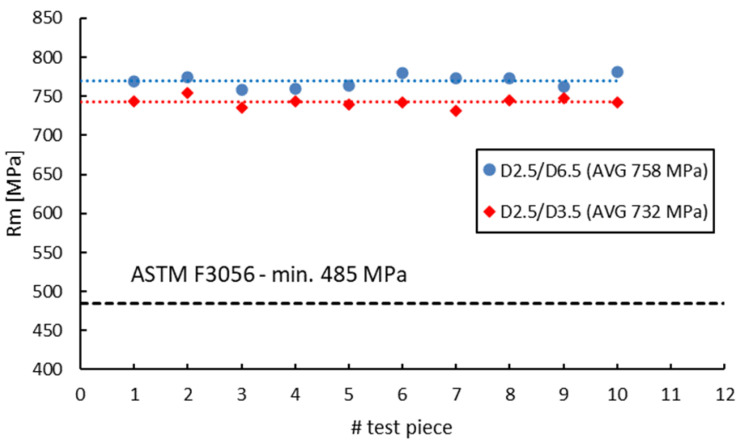
Tensile strength of 2.5 mm gauge diameter test specimens.

**Figure 19:**
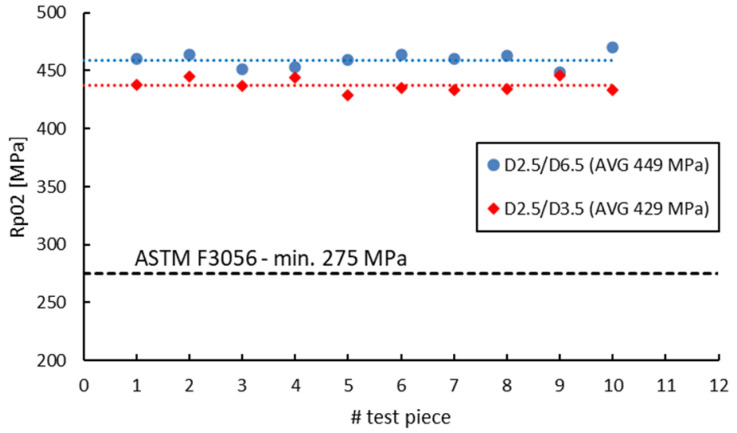
Yield strength of 2.5 mm gauge diameter test specimens.

**Figure 20:**
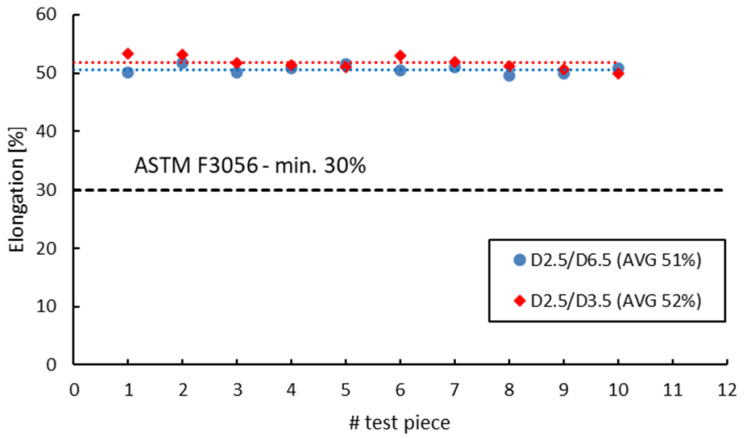
Elongation after fracture of 2.5 mm gauge diameter test specimens.

**Figure 21:**
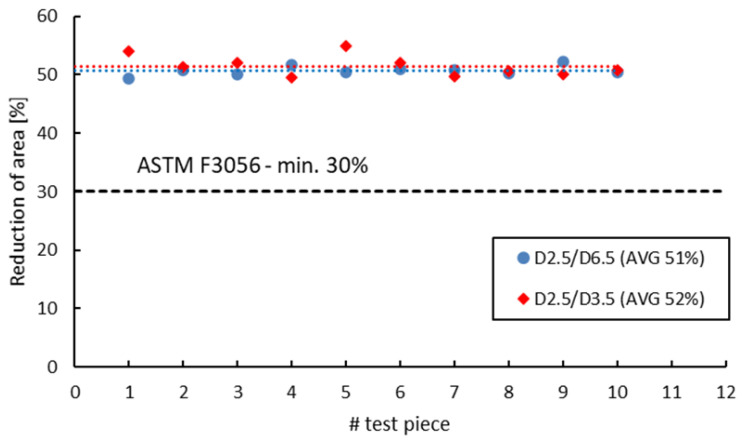
Reduction in area of 2.5 mm gauge diameter test specimens.

Figure 18, Figure 19, Figure 20 and Figure 21 compare the tensile test results obtained from 2.5 mm gauge diameter sub-sized tensile test specimens machined from coupons with different diameters, 6.5 mm and 3.5 mm, respectively. Test specimens no. 1 to 5 of both series were built together in the first job and test specimens no. 6 to 10 were built together in the second job. The black dotted lines in the figures represent the minimum mechanical property requirements according to ASTM F3056 [41], while the red and blue dotted lines represent the average values of all test pieces.

Similar to the 3 mm gauge diameter sub-sized test specimens, the tensile test results obtained from 2.5 mm gauge diameter sub-sized test specimens show higher tensile strength and yield strength values for specimens manufactured from 6.5 mm diameter coupons compared to those manufactured from 3.5 mm diameter coupons. Moreover, the elongation after fracture and reduction in area present in this case also have similar or much-closed values. Table 12 presents the values of the mechanical properties obtained from the 3 mm and 2.5 mm gauge diameter specimens manufactured from the two types of coupons. The previously obtained results on standard specimens (D5/D10), as well as on 3 mm gauge diameter test specimens (D3/D10) machined from 10 mm coupons (built in the first job) as the standard specimens are also included for discussions.

The analysis of the results presented in Table 12 shows that the tensile strength and yield strength of sub-sized specimens are lower than those obtained on standard specimens. Even the results obtained on 3 mm diameter sub-sized test specimens machined from the same diameter coupons (10 mm) (D3/D10) as the standard test pieces are lower, showing the “size effect” on mechanical properties. These specimens (D3/D10) exhibit comparable values with those obtained on test pieces machined from 6.5 diameter coupons, the subject of this study.

**Figure 22:**
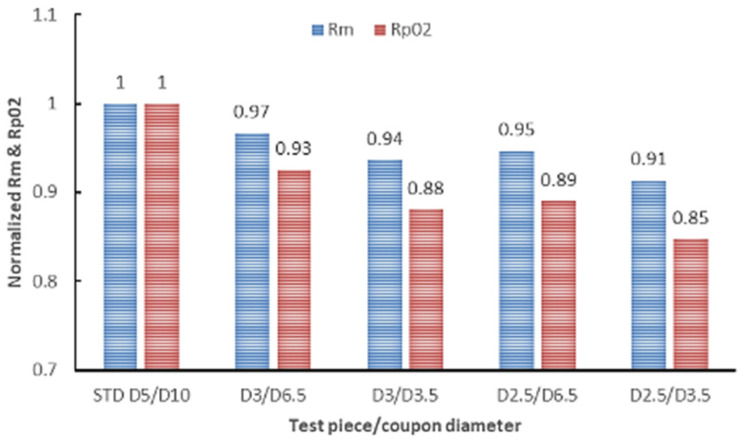
Normalized strength of sub-sized to standard test specimens.

**Figure 23:**
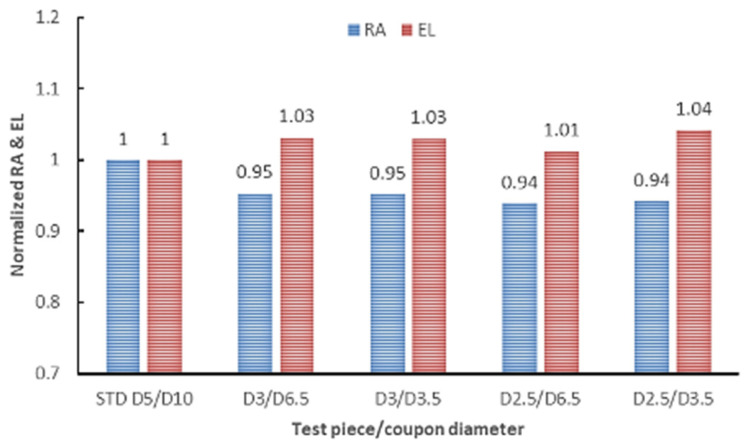
Normalized RA and elongation of sub-sized to standard test specimens.

To conduct an overall analysis, all test results obtained on sub-sized test pieces in this study were normalized to standard specimens’ results. The normalized values are presented in Figure 22 for UTS and YS and Figure 23 for elongation and RA.

The comparative analysis of the results shown in Figure 22 indicates that the strength (both UTS and YS) of the sub-sized test specimens is influenced by the test specimen gauge diameter and the diameter (thickness) of the coupon from which they were machined. Regardless of the coupon and specimen gauge diameters, the normalized reduction in area of sub-sized specimens is lower than that of standard specimens, while the elongation is a few per cent higher. The higher elongation is consistent with other authors’ findings. Yang et al. [78] showed that the ductility of the miniature specimen of 1.25Cr-0.5Mo steel is much higher than that of the standard specimen. They attributed the higher ductility of the miniature specimens to the void growth. They also showed that the fracture is caused by the cleavage under a multiaxial stress state and showed the specimen geometry’s critical role during the post-necking elongation. Dzugan et al. [58] confirmed the effectiveness of miniature sample testing for AM part as a production check, but they also revealed that the small thickness regions within a manufactured part can exhibit significant differences in tensile behaviour compared to thicker regions. In the absence of a clear method to determine the applicability and validity of coupon testing for a specific type of component, and considering the uncertainties surrounding the reproducibility of the printing process, it is challenging to assess how well a coupon will represent a final part. However, the mechanical properties measured in this study on sub-sized test specimens meet the minimum requirements for IN 625 AM material, as specified in ASTM F3056 [41]. Interestingly, the results obtained in our testing campaign contradict findings from several studies conducted on different materials, machines, or technology. Karnati et al. [79] fabricated and tested miniature tensile specimens using LBPF from 304L stainless steel and found that varying the gage length had no impact on the yield and tensile strength. The lower strength (both UTS and YS) observed in the sub-sized test specimens compared to standard-sized tensile specimens can be attributed to the size effect of the test pieces. Similarly, the variation in elongation measurements between the two types of test specimens (standard and sub-sized) are also attributed to the size effects. This effect is also presented in the literature for other materials [54,78,80]. Another explanation for these differences may be related to the thermal exposure that is different for sub-sized and standard specimens produced by laser-based metal additive manufacturing. Two major observations can be made regarding the overall analysis of tensile test results on sub-sized test specimens of AM IN 625:

- Sub-sized test specimens machined from the same type of coupon (cylindrical or profiled) exhibit lower strength as the gauge diameter decreases (2.5 mm);
- Sub-sized test specimens machined from cylindrical and profiled coupons with the same gauge diameter (2.5 mm or 3 mm) show lower strength when machined from a lower diameter coupon (3.5 mm) compared to a higher diameter coupon (6.5 mm), which were built and heat-treated together under the same conditions.

All of these findings lead to the conclusion that the behaviour of sub-sized test specimens can be influenced not only by size considerations but also by test specimen roughness or other intrinsic material properties. Additionally, the layer-by-layer nature of the L-PBF process makes the manufactured materials highly dependent on various factors. On one hand, the material properties can be significantly influenced by the printing conditions, machine, and powder type used. On the other hand, the processing and post-processing operations also play an important role in the material’s characteristics. It is also not to be ignored the potential impact of the equipment type, testing method/conditions and operator’s measurement or interpretation on the overall assessment of the material, irrespective of the technology used in its production. Sergueeva et al. [80] concluded that the tensile results obtained from specimens with different gage lengths cut from thin polycrystalline sheets and amorphous Fe-based ribbons depend not only on the property of the material itself and testing conditions but also on specimen size, geometry and even by the amorphous or crystalline materials. Therefore, it is crucial to consider the material’s microstructural characteristics developed during manufacturing, post-processing and testing when addressing the tensile behaviour of sub-sized specimens.

In the absence of a clear method to determine the applicability and validity of coupon testing for a specific type of component, and considering the uncertainties surrounding the reproducibility of the printing process, it is challenging to assess how well a coupon will represent a final part. However, our study emphasizes the significance of adapting characterization methods to the unique characteristics of manufactured parts. This includes considering reduced thicknesses that render sampling standard-size specimens impractical.

## 5. Conclusions

The tensile properties of AMed IN 625 were assessed using nonstandard, sub-sized tensile specimens with a gauge diameter of 2.5 mm and 3 mm. The primary purpose of using these miniaturized specimens is to significantly reduce material and time consumption while minimizing the amount of raw material needed. Additionally, these miniaturized specimens are particularly relevant for manufacturing parts with smaller wall thicknesses compared to standard tensile test pieces.

These sub-sized specimens were machined from three types of coupons manufactured on the *Z*-axis, where the standard test pieces showed the lowest strength. To facilitate the testing of the nonstandard specimens, a special fixing device was manufactured.

Regardless of the gauge diameter (2.5 mm or 3 mm), all sub-sized test specimens show tensile strength and yield strength lower compared to the values obtained on standard test specimens.

Sub-sized test specimens manufactured from 6.5 mm diameter coupons exhibit for both gauge diameters (2.5 mm or 3 mm) higher tensile strength and yield strength compared to the test specimens manufactured with the same gauge diameters but from profiled 3.5 mm diameter coupons. Additionally, both elongation after fracture and reduction in area present similar average values.

The results obtained on specimens with different diameters (2.5 mm and 3 mm) from the same type of coupon (6.5 mm or 3.5 mm) show that the lower the gauge diameter, the lower the recorded strength.

Regardless of the coupon and specimen gauge diameter, the normalized reduction in the area of sub-sized test specimens is lower than that of standard specimens, while the elongation is a few percentages higher. In terms of percentage, when comparing the tensile strength of sub-sized specimens with the standard specimens machined from 10 mm coupons (D3/D10), the decreases are as follows: D3/D6.5 (3%), D3/D6.5 (3%), D3/D3.5 (7%), D2.5/D6.5 (7%), and D2.5/D3.5 (9%). More significant decreases were found in the case of yield strength: D3/D65 (7%), D3/D6.5 (8%), D3/D3.5 (12%), D2.5/D6.5 (14%), and D2.5/D3.5 (18%). As for reduction area and elongation, the values obtained for the sub-sized specimens were consistently approximately 6% lower higher compared to the standard specimens. In contrast, the elongation values of the sub-sized specimens were approximately 2% higher compared to the standard specimens. However, the tensile test results of sub-sized test specimens still exceed the minimum values specified in the active Standard.

The main reason for the lower strength (both tensile strength and yield strength) compared to standard test specimens has been assigned to the “size effect” of the test specimens. Likewise, the differences between the measured elongations on the two types of test specimens (standardised and sub-sized) are attributed to this effect too. This observation can be attributed to the fact that a smaller specimen diameter with the same grain size provides fewer grain boundaries to impede dislocation motion, potentially affecting the material’s deformation behaviour.

Based on the overall analysis and results, the study concluded that in the case of L-PBF-manufactured IN 625, the SSTT can be used for quality control of the material and to experimentally verify it is with the minimum requirements of AMed IN 625 tensile properties. However, it is important to consider that the reduction in specimen size should be carried out in a way that preserves the representative nature of the mechanical properties of the bulk material.

## Figures and Tables

**Table 1:** Tensile test results of 3 mm gauge diameter test specimens machined from 6.5 mm diameter coupons (D3/D6.5)—manufactured during the first job.

Test Specimen #	M1	M2	M3	M4	M5	M6	M7	AVG	STDEV
UTS [MPa]	797	787	789	786	790	782	765	785	±10.0
YS [MPa]	491	487	478	474	487	476	460	479	±10.6
RA [%]	50	50	52	50	53	54	51	52	±1.8
Elongation [%]	49	51	53	52	51	51	53	51	±1.3

**Table 2:** Tensile test results of 3 mm gauge diameter test specimens machined from 6.5 mm diameter coupons (D3/D6.5)—manufactured during the second job.

Test Specimen #	M1	M2	M3	M4	M5	M6	M7 *	AVG	STDEV
UTS [MPa]	792	784	783	797	784	781	-	787	±6.2
YS [MPa]	484	474	473	N/R	471	473	-	475	±5.1
RA [%]	52	50	51	51	51	53	-	51	±1.2
Elongation [%]	53	52	53	50	52	51	-	52	±1.1

* scrapped during machining.

**Table 3:** Tensile test results of 3 mm gauge diameter test specimens machined from 3.5 mm diameter coupons (D3/D3.5)—manufactured during the first job.

Test Specimen #	M1	M2	M3	M4	M5	M6	M7	AVG	STDEV
UTS [MPa]	765	764	763	757	764	763	751	761	±5.1
YS [MPa]	460	456	460	452	459	460	448	456	±4.8
RA [%]	55	53	52	50	51	51	50	52	±1.8
Elongation [%]	53	54	50	51	51	51	51	52	±1.3

**Table 4:** Tensile test results of 3 mm gauge diameter test specimens machined from 3.5 mm diameter profiled coupons (D3/D3.5)—manufactured during the second job.

Test Specimen #	M1	M2	M3	M4	M5	M6	M7	AVG	STDEV
UTS [MPa]	760	766	755	767	761	765	754	761	±5.2
YS [MPa]	454	452	446	446	461	458	450	452	±5.7
RA [%]	49	50	52	52	54	51	50	51	±1.4
Elongation [%]	53	50	53	53	51	50	50	51	±1.5

**Table 5:** Tensile test results of 2.5 mm gauge diameter test specimens machined from 6.5 mm diameter coupons (D2.5/D6.5)—manufactured during the first job.

Test Specimen #	M8	M9	M10	M11	M12	AVG	STDEV
UTS [MPa]	769	775	758	760	764	765	±6.9
YS [MPa]	460	464	451	453	459	457	±5.3
RA [%]	49	51	50	52	50	50	±0.9
Elongation [%]	50	52	50	51	52	51	±0.8

**Table 6:** Tensile test results of 2.5 mm gauge diameter test specimens machined from 6.5 mm diameter coupons (D2.5/D6.5)—manufactured during the second job.

Test Specimen #	M8	M9	M10	M11	M12	AVG	STDEV
UTS [MPa]	780	773	773	762	781	774	±7.6
YS [MPa]	464	460	463	449	470	461	±7.7
RA [%]	51	51	50	52	50	51	±0.7
Elongation [%]	50	51	50	50	51	50	±0.6

**Table 7:** Tensile test results of 2.5 mm gauge diameter test specimens machined from 3.5 mm diameter profiled coupons (D2.5/D3.5)—manufactured during the first job.

Test Specimen #	M8	M9	M10	M11	M12	AVG	STDEV
UTS [MPa]	743	755	735	744	740	743	±7.4
YS [MPa]	438	445	437	444	429	439	±6.4
RA [%]	54	51	52	50	55	52	±2.2
Elongation [%]	53	53	52	51	51	52	±1.1

**Table 8:** Tensile test results of 2.5 mm gauge diameter test specimens machined from 3.5 mm diameter profiled coupons (D2.5/D3.5)—manufactured during the second job.

Test Specimen #	M8	M9	M10	M11	M12	AVG	STDEV
UTS [MPa]	742	732	745	748	742	742	±6.0
YS [MPa]	435	433	434	446	433	436	±5.5
RA [%]	52	50	51	50	51	51	±0.9
Elongation [%]	53	52	51	51	50	51	±1.1

**Table 9:** Corrected UTS—Test specimens with 3 mm gauge diameter machined from 6.5 mm diameter coupons (manufactured during the first job).

Test Specimen #	M1	M2	M3	M4	M5	M6	M7	AVG
Measured do, mm	3.031	3.000	3.010	2.998	3.013	3.040	3.018	-
Corrected deff, mm	3.023	2.992	3.002	2.990	3.004	3.032	3.010	-
Max. force, N	5752	5562	5614	5549	5631	5677	5473	-
Measured UTS, MPa	797	787	789	786	790	782	765	785
Corrected UTS, MPa	801	791	793	790	794	786	769	789

**Table 10:** Corrected UTS—Test specimens with 2.5 mm gauge diameter machined from 6.5 mm diameter coupons (manufactured during the first job).

Test Specimen #	M8	M9	M10	M11	M12	AVG
Measured do, mm	2.512	2.502	2.518	2.508	2.517	-
Corrected deff, mm	2.503	2.493	2.510	2.499	2.509	-
Max. force, N	3810	3809	3774	3753	3802	-
Measured UTS, MPa	769	775	758	760	764	785
Corrected UTS, MPa	774	780	763	765	769	789

**Table 11:** Dimensionless parameter calculated for the sub-sized test specimens.

Test Specimen #	D3/D6.5	D2.5/D6.5	D3/D3.5	D2.5/D3.5
UTS [MPa]	786	770	761	743
YS [MPa]	477	459	454	437
*Gs*, μm	0.055	0.055	0.063	0.063
*So*, μm^2^	7.069	4.909	7.069	4.909
*Ssl* μm	2.659	2.216	2.659	2.216
*lgr*	262	183	230	160
*lgr*/*Ssl*	99	83	86	72

**Table 12:** Average values of tensile test results obtained on different gauge diameter test specimens.

Test Specimen #	D5/D10 *	D3/D10	D3/D6.5	D3/D3.5	D2.5/D6.5	D2.5/D3.5
UTS [MPa]	813	786	786	761	770	743
YS [MPa]	516	483	477	454	459	437
RA [%]	54	51	51	51	51	51
Elongation [%]	50	52	52	52	51	52

* Standard specimens.

## Data Availability

Not applicable.
